# Hybrid DFT Quality Thermochemistry and Environment Effects at GGA Cost via Local Quantum Embedding

**DOI:** 10.1021/acs.jctc.5c01121

**Published:** 2025-09-29

**Authors:** József Csóka, Dénes Berta, Péter R. Nagy

**Affiliations:** † Department of Physical Chemistry and Materials Science, Faculty of Chemical Technology and Biotechnology, 61810 Budapest University of Technology and Economics, Müegyetem rkp. 3., H-1111 Budapest, Hungary; ‡ HUN-REN−BME Quantum Chemistry Research Group, Müegyetem rkp. 3., H-1111 Budapest, Hungary; § MTA−BME Lendület Quantum Chemistry Research Group, Müegyetem rkp. 3., H-1111 Budapest, Hungary

## Abstract

Reliable thermochemical modeling of reaction mechanisms requires hybrid DFT or higher-level models as well as inclusion of environment, conformer, thermal, etc. effects. Quantum embedding, such as the Huzinaga-equation and projection-based models employed here, can make such computations more accessible by focusing the use of the more costly models to the atoms involved in forming and breaking the bonds or residing in interacting surfaces, etc. Here, we further accelerate these embedding computations by combining local approximations in the atomic orbital and auxiliary function space of the hybrid DFT part with a new in-core density fitting implementation optimized for multilayer DFT. The so introduced local embedded subsystem (LESS) framework, when increasing the size of the environment, leads to asymptotically constant cost for the hybrid DFT layer. We demonstrate on reaction and activation energies of practical homogeneous, heterogeneous and enzymatic catalysis reactions that the intrinsic accuracy of hybrid DFT is retained, with a few tenths of a kcal/mol error including all (embedding and local) approximations. Compared to the same complete (density fitted) hybrid DFT reference, the LESS hybrid DFT-in-GGA runtimes are 30–90 times faster on systems with up to 171–238 atoms. Achieving energetics with practically hybrid DFT quality and GGA cost is a significant step toward predictive thermochemistry including reliable sampling, dynamics, etc. as well as quantum environment effects.

## Graphical abstract

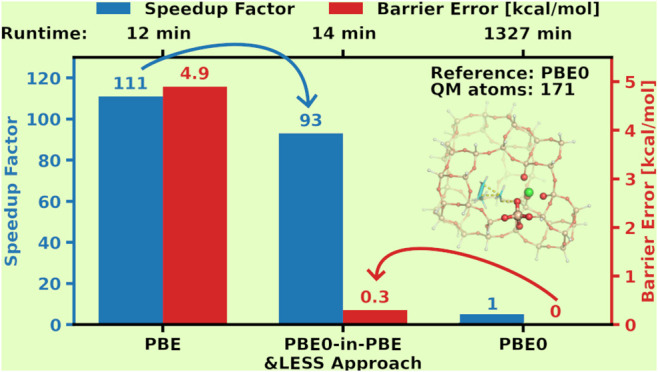

## 1 Introduction

Electronic structure calculations are required and thus routinely employed to model a large variety of chemical properties, including molecular interactions, catalytic reaction mechanisms, light-matter interactions, etc. However, accurate simulations incorporating, e.g., thermochemical and environment effects in homogeneous, heterogeneous or enzyme reactions require the quantum mechanical (QM) treatment of up to a few hundred atoms. This poses a limitation on our most accurate electronic structure methods suitable for reactivity modeling, especially when the exploration of the conformational space or dynamic properties is also of interest. Moreover, chemically accurate modeling of thermodynamic and especially kinetic properties often requires models beyond the capabilities of pure density functional theory (DFT) models. Although efficient local correlation based wave function approaches, such as the local natural orbital (LNO) based and other coupled cluster (CC) methods can now routinely scale up to a few hundred atoms,^1–3^ their application is at the moment limited to single point energy calculations. Hence, DFT remains the dominating electronic structure method due to its affordable computational cost and the availability of a wide range of properties beyond energetics. However, the accurate modeling of chemical reactions often require hybrid (or higher rung) functionals and sufficiently large basis sets.^4,5^ These methods have a computational cost that often necessitates compromises on the size of the chemical model system, the treatment of its environment or the quality of thermochemical corrections.

Multilevel modeling techniques are popular tools to reduce the computational cost by combining different theoretical methods for the subsystem in focus and its environment. For example, QM/MM^6–8^ methods have a pivotal role in describing chemical processes in biochemical,^9,10^ material,^11,12^ surface,^13^ cavity,^14^ explicit solvent,^15^ etc. environments. To improve the quality of the environment model or to decrease the size of the high-level QM model, multilevel QM-QM embedding methods are also available. These algorithms divide the QM system into two or more subsystems, which are modeled with different QM models, such as via different rungs of DFT functionals, semiempirical QM models or even wave function approaches. Typically, a more accurate level of theory is used for the chemically active region (describing bonds breaking and forming) and a faster method is applied for the environment regions (e.g., solvent, protein structure, surface of solids).

The family of QM-QM methods include ONIOM,^16–18^ density matrix embedding,^19^ embedded mean-field theory,^20,21^ frozen density embedding,^22–24^ subsystem DFT,^25–27^ fragmentation methods,^28–32^ orthogonality constrained basis set expansion,^33^ wave function-in-wave function embedding,^1,34–38^ reduced density matrix embedding.^39^ Here, we focus on the projection-based^40–44^ and the numerically very similar, but formally exact Huzinaga-equation-based embedding methods.^43,45,46^

The Huzinaga and projector embedding schemes divide the system into two parts, an active region and an environment. The active region is typically described with a (double) hybrid DFT functional or a wave function method. In both cases, a computationally demanding self-consistent (SCF) field calculation [Hartree–Fock (HF) or hybrid DFT] is performed on the active subsystem. For hybrid DFT level high-level calculations, the performance bottleneck is the exact exchange term in the Fock (or Kohn–Sham) matrix, and thus our goal here is to accelerate its computation.

In four-center integral based hybrid DFT (or HF) algorithms, the Coulomb and exchange matrices are built using the density matrix expanded in atomic orbital (AO) basis. In their original form, projection and Huzinaga based DFT-in-DFT embedding schemes do not reduce the number of AO functions in the high-level calculation, thus, the high-level method remains fourth-order scaling with the size of the complete QM system. One direction of improvement on the high-level execution times could be the use of accelerated HF and hybrid DFT approaches. For example, advanced screening^47–49^ and incremental SCF^50–52^ methods can greatly reduce the number of integral evaluations and contractions. Alternatively, seminumerical methods perform one of the integration numerically and the other analytically to compute the four-center two-electron integrals, including the pseudospectral method,^53–57^ chain of spheres exchange,^58–61^ and other low-scaling seminumerical^62,63^ approaches. Moreover, the auxiliary density matrix method^64,65^ and tensor factorization ideas,^66,67^ including tensor hypercontraction^68,69^ and the multipole approaches^70–76^ are also effective. Here, we employ density fitting (DF),^77–82^ which can be used to expand the AO product densities on an auxiliary (or fitting) basis. Although DF does not change the theoretical scaling of the SCF algorithm (with exact exchange), in practice, it can be significantly faster than four-center algorithms, especially with reliable AO basis sets. The DF scheme can be made even more efficient, for example, via (pair) atomic resolution of identity (RI) approaches,^83,84^ occupied-orbital RI,^85^ or local DF methods,^86–89^ the latter employed also in the present work.

Although the above methods are efficient and generally applicable to speed up SCF calculations, they are not yet combined with the ideas exploited in embedding methods, such as partitioning to low- and high-level subsystems. The first step along these lines within the projection-based and Huzinaga embedding schemes was the development of approximations to decrease the size of the AO basis. A dual basis set approach was combined with embedding by Hégely and co-workers,^45^ an AO basis set reduction scheme was published by Barnes and co-workers^90^ and was later further developed by Bennie et al.^91^ and Bensberg and Neugebauer.^92^ In AO basis reduction, one exploits that only a subset of the AOs are required for the accurate expansion of the active MOs, as shown also in various chemical applications.^93–96^ This reduces the scaling of the high-level calculations, which, at least for four-center integral based methods, could become (asymptotically) independent of the size of the full system.

Our aim is to extend the use of reduced-cost and embedding approaches beyond four-center algorithms, i.e., in combination with DF. Already without additional approximations, DF-SCF benefits from embedding as the cost of the exchange matrix computation is proportional to the number of active MOs, thereby reducing the overall scaling to third order. Furthermore, here we introduce the new local embedded subsystem (LESS) framework, employing AO and new DF auxiliary basis set reduction approaches for the active subsystem. First, extending local AO reduction to DF-SCF decrease its scaling already to quadratic, due to the quadratic scaling of some of the rate-determining steps of DF-SCF with the number of DF auxiliary functions. Second, we generalize this local AO approximation to DF auxiliary basis reduction in the high-level calculation. Combining these two developments, the computer time needed for the high-level DF-SCF calculation does not depend on the size of the full system in the limit of much larger environments.

An additional advantage of the new LESS method is that the combined reduction on both the AO and DF auxiliary bases makes it possible to store the three-center DF electron-repulsion integrals (ERI) in the primary memory, even for hundreds of atoms using at least triple-ζ quality basis sets. Note that, storing the integrals in standard four-center and DF algorithms would not be possible due to the size of the ERI tensor, highlighting the benefits of combining LESS and DF-SCF. To utilize this, we also developed an efficient in-core DF-SCF algorithm, which reduces the runtime by a factor of 4–5 compared to the integral-direct implementation. The combined approximations still have low errors in the few tenths of a kcal/mol range, which does not alter the intrinsic accuracy of hybrid DFT methods. However, the DFT-in-DFT computations using the LESS, DF-based and in-core developments lead to only about twice as long computation times as required by pure, DF accelerated GGA rung DFT models. The achieved accuracy and efficiency properties are demonstrated on real-world applications, including biochemical, organo- and surface catalytic reactions with realistic environments of about 200 QM atoms. Reaching DF-GGA level speed represents a significant step toward efficient conformational sampling and dynamics studies in accurate reactivity predictions, including reliable environment models.

In this article, in section 2, we start by presenting the theory of the Huzinaga and projection-based embedding methods as well as the new algorithms for the atomic and auxiliary basis reduction. Section 3 details the representative chemical reactions on which the methods accuracy (section 4) and computational affordability (section 5) are demonstrated. Finally, in section 6, we present discussion and general advice for applying the LESS framework in practical computational chemistry.

## 2 Theory

### 2.1 Huzinaga- and Projection-Based Embedding

In this section, we give an overview of the Huzinaga-equation-based^43^ and the projector embedding^41^ methods. Both methods determine the low- and high-level models and a set of atoms constituting the active region similarly. In the first step, the low-level calculation is performed for the whole QM system. Next, the low-level occupied molecular orbitals (MOs) are localized. The MOs residing on the subsystem atoms are selected as active MOs and the rest constitute the environment orbitals. The MO selection can be performed in several ways, for example, by using the Mulliken population based^41,43^ or the SPADE algorithm.^97,98^ In the last step, the high-level MOs are optimized with the high-level SCF method, while the environment MOs are kept frozen. In order to avoid the use of nonadditive kinetic energy potential, the high-level MOs are made to be orthogonal to the environment orbitals. This can be achieved by a level-shift operator^41^ as used in projector embedding or by the adoption of the Huzinaga equations,^43,99^ which we used in this work. In brief, the Huzinaga equations result from the optimization of a single determinant with the side conditions enforcing the orthogonality of the active MOs to the environment MOs. Note that wave function method-based correlation calculation can also be executed for the active subsystem (after a Hartree–Fock calculation) and even multiple levels of LNO CC layers can be embedded into DFT environments.^43,45^

The total energy for DFT-in-DFT embedding is written as

1 $$E=E_{\mathrm{LL}}\left[D^{\mathrm{AB}}\right]-E_{\mathrm{LL}}\left[D^{A}\right]+E_{\mathrm{HL}}\left[{\mathrm{D̃}}^{A}\right]+\mathrm{Tr}\left\{\left({\mathrm{D̃}}^{A}-D^{A}\right)\frac{\partial E_{\mathrm{LL}-\mathrm{HL}}}{\partial D^{A}}\right\}$$

where LL and HL indices are used for the low- and high-level calculations, respectively, while *AB*, *A* and *B* label the full, active and environment systems, respectively. Moreover, ***D* ~**^*A*^ denotes the subsystem density calculated with the high-level method and

2 $$E_{\mathrm{LL}-\mathrm{HL}}=E_{\mathrm{LL}}\left[D^{\mathrm{AB}}\right]-E_{\mathrm{LL}}\left[D^{A}\right]+E_{\mathrm{HL}}\left[{\mathrm{D̃}}^{A}\right]$$

The orthogonality of the high-level subsystem and environment MOs can be enforced by either a level-shift operator in projector embedding or the Huzinaga equations. In the case of projector embedding, a projector of

3 $$P_{\mu \nu }=\sum_{\rho \sigma }S_{\mu \rho }D_{\rho \sigma }^{B}S_{\sigma \nu }$$

is added to the Fock or Kohn–Sham matrix as

4 $${\mathrm{F̃}}_{\mu \nu }+\alpha P_{\mu \nu }$$

Here ***S*** is the AO overlap, α is the level-shift and the high-level Fock matrix is defined as

5 $${\mathrm{F̃}}_{\mu \nu }=\frac{\partial E}{\partial {\mathrm{D̃}}_{\mu \nu }^{A}}$$

In the α → ∞ limit, the projector based embedding becomes exact. In practice, only finite α values can be used, which have an often negligible effect on the total energy but it may be more difficult to handle for other properties.

The use of the finite level-shift can be avoided by adding orthogonality constraints to the Lagrangian of the high-level SCF method. The effective Kohn–Sham matrix of the high-level SCF (called the Huzinaga matrix) can be derived from this Lagrangian^99,100^ and reads as

6 $$H_{\mu \nu }={\mathrm{F̃}}_{\mu \nu }-\sum_{\rho \sigma }S_{\mu \rho }D_{\rho \sigma }^{B}{\mathrm{F̃}}_{\sigma \nu }-\sum_{\rho \sigma }{\mathrm{F̃}}_{\mu \rho }D_{\rho \sigma }^{B}S_{\sigma \nu }$$

The SCF procedure is performed as usual with ***H*** at the place of the Fock or Kohn–Sham matrix.

In general, the high-level SCF calculation is the rate-determining step during the energy evaluation due to the two-electron part (especially the exact exchange contribution) of the Kohn–Sham matrix. With density fitting, the two-electron contribution reads as

7 $$G_{\mu \nu }\left[{\mathrm{D̃}}^{A}\right]=\sum_{\sigma \lambda \mathrm{PQ}}{\mathrm{D̃}}_{\lambda \sigma }^{A}\left(\mu \nu |P\right){\left(P|Q\right)}^{-1}\left(Q|\lambda \sigma \right)-c_{\mathrm{HFx}}\sum_{i\sigma \lambda \mathrm{PQ}}{\mathrm{C̃}}_{\sigma i}^{A}{\mathrm{C̃}}_{\lambda i}^{A}\left(\mu \lambda |P\right){\left(P|Q\right)}^{-1}\left(Q|\sigma \nu \right)$$

where the Greek letters denote the AOs, *P*, *Q* are fitting basis functions, *c*_HFx_ is the weight of the Hartree–Fock exchange contribution and ***C* ~**^*A*^ is the high-level MO coefficient matrix. The construction of the exchange matrix [second term in eq 7] takes advantage of the embedding approximation as the MO index *i* is restricted to the active orbitals, and thus its overall scaling is reduced to third power. Further savings can be achieved by introducing local approximations to reduce the AO basis, which improves the scaling to second-order by limiting the AO indices (μ, ν, λ, σ) in eq 7. In addition, in the LESS framework we introduce new algorithms, which retain the most important auxiliary functions and thus restrict the summation over the fitting functions (*P*, *Q*). This way, the operation count of the Coulomb and exchange matrix formation becomes asymptotically independent of the full system size, and depends only on the size of the active subsystem.

### 2.2 Reduction of the Atomic Orbital Basis

We can exploit that only a small set of AOs is significant for the expansion of the active MOs.^90^ The selection of the important functions is based on the net Mulliken population^91^

8 $$q_{\mu }=\sum_{i}C_{\mu i}^{A}C_{\mu i}^{A}S_{\mu \mu }$$

where ***C*** ^*A*^ is the low-level active subsystem MO coefficients and ***S*** is the AO overlap. The basis function μ is kept in the high-level calculation if *q*_μ_ > ε_AO_, where ε_AO_ is a predefined threshold. It is important to note that if a single function is retained from a given shell then the other functions of the shell are also kept.

The low-level SCF is solved in the complete AO basis. If any AOs are dropped after this point, the AO expansion of the low-level MOs is also truncated, but only when they are used to build the embedding potential or projector in eqs 6 and 3, respectively. After the AO basis is reduced, the orthogonality of these low-level MOs are lost. To maintain consistency in the high-level calculation, both the environment and the active low-level orbitals are (re)­orthogonalized in the reduced AO metric (separately from each other), using Löwdin canonical orthogonalization. Namely, the overlap of the MOs in the reduced basis is diagonalized and the MOs are transformed with the eigenvectors. As the retained AOs are concentrated around the active subsystem, some of the environment MOs far from the active atoms may not be accurately expanded in the reduced AO basis. These MOs can be detected via their small MO overlap eigenvalues and then they are removed from the space of the environment orbitals. Next, to restore the orthogonality of the environment and all active MOs, the subspace of the retained environment MOs is projected out from all active orbitals.

### 2.3 Reduction of the DF Auxiliary Function Basis

Here, we propose three alternative methods for the reduction of the DF auxiliary function basis for the high-level calculation. First, a straightforward way to construct a reduced fitting basis set is the use of natural auxiliary functions (NAFs).^101^ The three-center integrals with NAFs in the place of the auxiliary functions is the best approximation to the original integral tensor in the least-squares sense.^101^ The NAF approach uses the singular value decomposition to construct this compressed representation. When Huzinaga embedding is combined with NAFs, first, the active low-level occupied MOs (***C*** ^*A*^) are used to build the

9 $$J_{\mu i}^{P}=\sum_{Q\nu }C_{\nu i}^{A}\left(\mu \nu |Q\right)L_{\mathrm{QP}}$$

half-transformed integrals, where ***L*** is the Cholesky factor of the inverse of the two-center Coulomb integrals. The right singular vectors (***N***) of ***J*** define the NAFs (optimized for the exchange matrix evaluation). In practice, the singular vectors and values are calculated as the eigendecomposition of *W*_*PQ*_ = ∑_μ*i*_ *J*_μ*i*_^*P*^ *J*_μ*i*_^*Q*^. The compressed integrals can be expressed as

10 $$J_{\mu \nu }^{\mathrm{P̅}}=\sum_{\mathrm{QR}}\left(\mu \nu |Q\right)L_{\mathrm{QR}}N_{R\mathrm{P̅}}$$

where *P̅* labels the retained NAFs corresponding to singular values larger than a threshold ε_NAF_.

In theory, the matrix ***W*** could be built from the AO integrals, however, it would result in a larger NAF space.^101^ The NAFs from the ***C*** ^*A*^-dependent integrals are a good choice, because ***C*** ^*A*^ and ***C* ~**^*A*^ can be expected to span a similar space. Alternatively, the NAFs could be determined from the high-level MOs (***C* ~**^*A*^) in each iteration of the high-level SCF, however, the overhead introduced by eqs 9 and 10 would be much larger than the performance gains. Consequently, the NAF method is not as well suited for integral-direct SCF algorithms either. Although the NAF approach yields the most compact compression of the auxiliary space, a potential drawback is that the calculation of the analytic gradients is also more complicated with the NAF approach.^102^

In our second approach, Mulliken-like charges were defined based on the analogy to the AO reduction. To define these generalized Mulliken charges, we exploit the analogy, that the NAFs are the system specifically optimal linear combination of the fitting functions, just like the MOs are the linear combination of the AOs. Thus, inspired by eq 8, the generalized Mulliken charges are defined as

11 $${\mathrm{q̂}}_{P}=\sum_{\mathrm{Q̅}}N_{P\mathrm{Q̅}}N_{P\mathrm{Q̅}}S_{\mathrm{PP}}^{\mathrm{DF}}$$

where ***S*** ^DF^ is the overlap of the auxiliary functions and *q̂*_*P*_ is the generalized Mulliken charge of the auxiliary function *P*. The fitting functions with *q̂* values above a threshold ε_Mull_ are retained, while the rest of the fitting functions are not used in the high-level computation. Similarly to the AO basis reduction, if an auxiliary function is kept in a shell then none of the shell’s functions are dropped. The advantage of this method is that it can be used together with integral-direct algorithms as the auxiliary functions are selected before the high-level calculation and the transformation step in eq 10 is not required. However, the computational overhead of the calculation and diagonalization of ***W*** is still present, even though it has to be performed only once.

In our third approach, a reduced fitting basis set is defined through the generalization of the local DF (LDF) domains used in LDF-SCF calculations.^86,87,103^ In the original LDF algorithm, a set of atoms (a domain) is selected specifically for each localized MO, and only the auxiliary functions centered on the selected atoms are used to fit the (μ*i*)-type orbital products in the exchange term, that is

12 $$\sum_{i}\sum_{P,Q\in \left[i\right]}\left(\mu i|P\right){\left(P|Q\right)}^{-1}\left(Q|\nu i\right)$$

where [*i*] is the domain of the *i*th MO and *P* ∈ [*i*] denotes that the summation runs over the auxiliary functions centered on the atoms in [*i*]. Our LDF-SCF algorithm^75,87^ could be used without any modification, however, it would not reduce the fitting basis set for the Coulomb term. Additionally, it would complicate the development of analytic derivatives^89^ as the LDF-SCF is not exactly variational due to the MO dependence of the second summation in eq 12.

To overcome these issues, we employ the fitting functions in the union of the domains to fit both the exchange and Coulomb terms. This way, the variational property of the SCF method is retained. Moreover, to increase the efficiency of our previous domain construction scheme,^75^ the domains are built from shells rather than atoms. First, Löwdin atomic charges are computed for each MO and atom. If the charge of an MO exceeds 0.05 on a given atom, then all shells of this atom are added to the so-called primary domain of the MO. This primary domain is then extended based on the Cauchy–Schwarz screening. We retain the fitting functions *P* centered on atom *B* with angular momentum l if

13 $$\max_{\mu \in A,\nu \in B}\sqrt{\left(\mu \nu |\mu \nu \right)}\max_{P\in B_{l}}\sqrt{\left(P|P\right)}>\varepsilon_{\mathrm{dom}}$$

holds for any atom *A* from the primary domain, where μ and ν are AOs on atoms *A* and *B* and ε_dom_ is a predefined threshold.

The LDF domain based fitting reduction is well suited for both integral-direct, disk-based and in-core SCF algorithms as the set of important fitting functions is built prior to the high-level algorithm using the low-level MO orbitals. Although not as compact as NAFs, this LDF based approximation is fast and introduces negligible overhead.

### 2.4 In-Core Algorithm

It is well-known that for large molecules and basis sets, it is not possible to store the three-center Coulomb integrals in the primary memory or even in the secondary memory (i.e., on disk). In such cases integral-direct approaches are used, which do not store but rather re-evaluate all required integrals in each SCF iteration. Our integral-direct DF-SCF implementation was presented in detail previously.^75^ However, by combining embedding methods with basis reduction and efficient screening, we can drastically reduce the number of integrals, and it becomes possible to store the integrals needed for the high-level computation in primary memory for practical applications.

In more details, let us use the notation

14 $$J_{\mu \nu }^{P}=\sum_{Q}\left(\mu \nu |Q\right)L_{\mathrm{QP}}$$

for the three-center Coulomb integrals. The Coulomb matrix ***F*** ^*J*^ is calculated in two steps

15 $$X_{P}=\sum_{\mu \nu }D_{\mu \nu }J_{\mu \nu }^{P}\;\mathrm{and}\;F_{\mu \nu }^{J}=\sum_{P}X_{P}J_{\mu \nu }^{P}$$

and similarly, the exchange matrix ***F*** ^*K*^ is built as

16 $$J_{\mu i}^{P}=\sum_{\nu }C_{\nu i}J_{\mu \nu }^{P}\;\mathrm{and}\;F_{\mu \nu }^{K}=-c_{\mathrm{HFx}}\sum_{\mathrm{iP}}J_{\mu i}^{P}J_{\nu i}^{P}$$

We optimize for the evaluation of the exchange matrix as this is the rate-determining step. Therefore, the *J*_*μν*_^*P*^ integrals are stored as follows: for a given shell *S* and AO function ν, the quantity

17 $$J_{\nu }^{S}=\max_{\mu \in S}\max_{P}J_{\mu \nu }^{P}$$

is calculated and if it is below threshold, the integrals are dropped. Otherwise, the integrals are stored in such an order that *P* is the fastest index, ν is the slowest and μ is in the middle restricted to *S*. Additionally, a mapping is stored which keeps a list of ν-S pairs for which *J*_ν_^*S*^ of eq 17 is above the screening threshold. The Coulomb and exchange matrices are evaluated shellwise, that is, the integrals for a given shell *S* are retrieved (either from primary memory or disk), the density and the MO coefficients are sorted according to the mapping and the first steps in eqs 15 and 16 are performed. Thus, the repeated recalculation of the integrals are avoided, moreover, the storage scheme makes it possible to use a single matrix-matrix and matrix-vector multiplication to get the *J*_*μi*_^*P*^ and *X*_*P*_ intermediates, respectively. Next, the second steps in eqs 15 and 16 are executed and the final result is sorted to the Fock or Kohn–Sham matrix. The implementation of the above in-core algorithm exploit the parallel processing power of modern CPUs by using shared memory parallel, optimized linear algebra subroutines and it does not rely on explicit parallelization.

We note that the main storage space savings, which make the in-core approach feasible, come from the AO and fitting basis reduction. The screening described above screens out a relatively small number of integrals (less than 30%) when combined with the basis set reduction schemes in LESS for typical systems, like those with up to 100–200 atoms considered here.

## 3 Chemical Applications

To assess the performance of our approximations, demonstrative reactions were investigated that are typically used to benchmark embedding methods:^20,43,45,92,104,105^ the deprotonation of decanoic acid, the hydrogenation of pentacene, the Diels–Alder reaction of octadecanonaene and 1,3-butadiene, the substitution reaction of 1-chlorodecane with a hydroxide anion to form 1-decanol and a chloride anion. The reactions are depicted in Figure 1.

**Figure 1:**
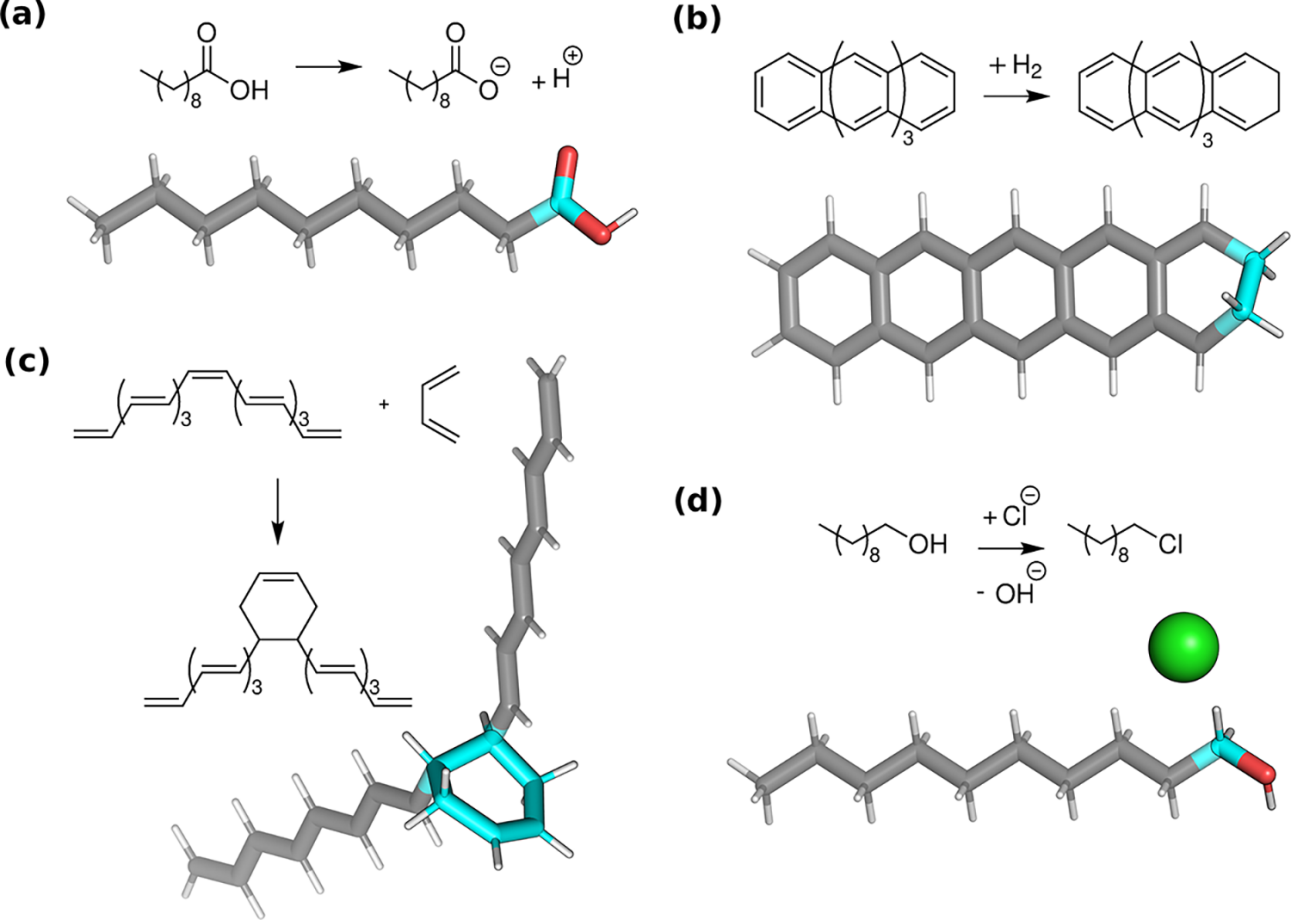
Reactions in standard tests. The active atoms are highlighted with color and can be found in the SI as well. (a) the deprotonation of decanoic acid (b) hydrogenation of pentacene (c) Diels–Alder reaction of octadecanonaene and 1,3-butadiene (d) substitution reaction of 1-chlorodecane with a hydroxide anion to form 1-decanol and a chloride anion.

Next, three larger test systems of 171–238 atoms were selected (see Figure 2). The first one is a zeolite catalyzed methylation reaction of propylene.^100,106^ It is known, that the reaction energy and the barrier are sensitive to the applied DFT functional and hybrid functionals are considerably more accurate.^106^

**Figure 2:**
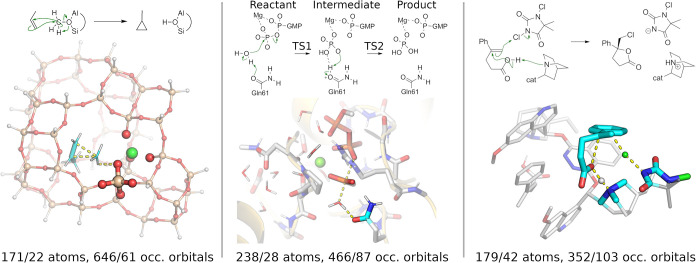
Zeolite catalyzed methylation reaction^106^ (left), enzymatic phosphate hydrolysis^107^ (middle), halocyclization reaction^111^ (right). The skeletal structures above show the reactions, while the figures below depict the selected QM environment (transparent) and active (solid colors) regions in the TS structures. The numbers on the bottom denote the total number of QM/active atoms and occupied orbitals.

Second, the GTP hydrolysis catalyzed by the KRas small GTPase activated by the p120-GAP is a typical example of phosphate biochemistry.^107^ The accurate description of the S_N_2-type phosphate cleavage requires hybrid DFT description,^108^ while calculated properties are notoriously slow to converge with the size of the QM region.^109,110^ Finally, the organocatalytic chlorolactonization involving a cationic chlorine transfer^111^ poses further challenge to GGA functionals,^112^ while the large organocatalyst required for stereoselectivity hinders the use of more expensive methods.

For the above reactions, (meta-)­GGA functionals typically yield too high errors. In the case of the enzymatic reaction,^108^ the GGA errors are in the −5.7 to −4.3 kcal/mol range. PBE^113^ is among the best at the (meta-)­GGA rungs, while PBE0 ^114^ is among the best hybrid and range-separated hybrid functionals compared to LNO coupled-cluster singles, doubles and perturbative triples [CCSD­(T)] ^1,2,115^ reference calculations. Therefore, we used the PBE0-in-PBE embedding method in our tests. For the zeolite-catalyzed reaction, the errors are between −9 to −15 kcal/mol with GGA functionals. The PBE family of functionals are among the best performers, thus we opted for the PBE0-in-PBE method again. In the case of the organocatalytic reaction, we found errors around −13.5 to −16.3 kcal/mol with GGA functionals with respect to LNO–CCSD­(T).^112^ At the meta-GGA rung, M06-L^116,117^ gave the best results with B97M-V^118^ being almost as good. Among the hybrid functionals, the CAM-B3LYP^119^ was the most accurate, thus we used CAM-B3LYP-in-M06-L embedding. For the small test reactions, the PBE0-in-PBE embedding was employed. We applied the D3 dispersion correction^120^ with Becke–Johnson damping^121^ for all reactions, both for the low- and high-level calculations.

Dunning’s cc-pV*X*Z (X = D, T, Q) basis set^122,123^ were used for the small and zeolite reactions with the corresponding cc-pV*X*Z-RI-JK^124^ fitting basis sets. For the other enzyme and organocatalytic reactions, a mixed basis set was used with def2-TZVP on the active and def2-SVP^125^ on the environment atoms. The fitting basis set was a mixed def2-QZVP-RI-JK^124^/def2-QZVP-RI-J^126^ for the enzyme reaction and def2-QZVP-RI-JK for the halocyclization as implemented in Mrcc.^127–129^ We used the AO reduction threshold of ε_AO_ = 10^–4^ unless stated otherwise.

The molecular structures of all test systems can be found in the Supporting Information (SI). The enzyme system was studied in a QM/MM setting. The MM point charges are also listed after the Cartesian coordinates, based on the CHARMM36m protein and TIP3P water force fields.^130,131^ All calculations were performed with the Mrcc program package.^127–129^ Our implementation uses OpenMP parallelization for all algorithms. Additionally, the Message Passing Interface (MPI) can also be used with integral direct calculations. The presented algorithms will be available in the next release free for academic use. A sample input file, additional timing, scaling, and numerical results are provided in the SI.

## 4 Accuracy

### 4.1 Standard Test Reactions

The effect of AO basis reduction was already benchmarked for these test reactions.^92^ Based on these results, we used the threshold value of ε_AO_ = 10^–4^, as it already gives reliable convergence and acceptable accuracy (around 1 kcal/mol). Thus, focusing on the new fitting basis reduction approach, we calculated the reaction energy errors of the auxiliary basis reduction with respect to the results obtained with AO reduction only. In our experience, the choice of basis set does not alter our results significantly (see the Figures S1 and S2 of the SI), therefore, only the errors corresponding to the cc-pVTZ basis is shown in detail in Figure 3.

**Figure 3:**
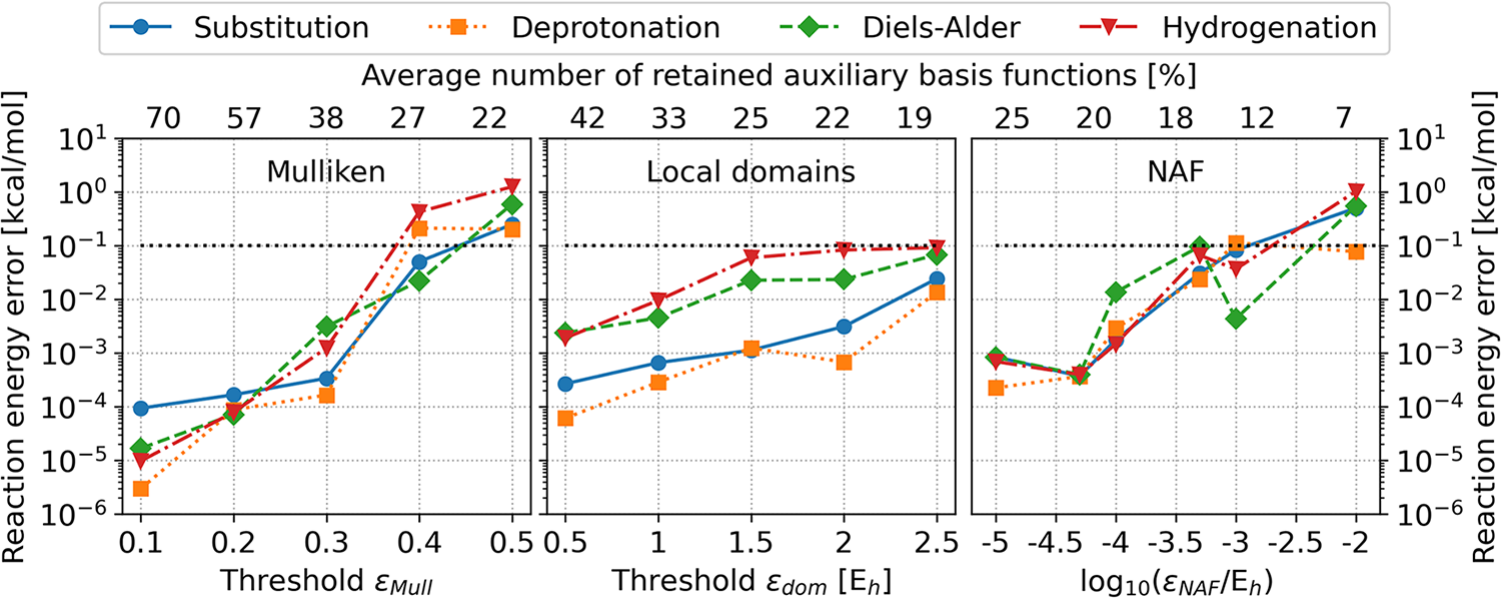
Reaction energy error of standard benchmark reactions (see text for details). The reference calculations use AO basis reduction only. The bottom *x* axis shows the applied thresholds, while the top *x* axis display the average ratio of kept fitting basis functions compared to the size of the complete auxiliary basis in %.

For all three tested fitting basis reduction methods, the errors are typically below 0.1 kcal/mol for affordably tight threshold values. The generalized Mulliken charges method retains around 40–70% of the fitting functions without notable loss in accuracy (errors are smaller than 0.01 kcal/mol) and around 30% of the fitting functions is enough if close to 0.1 kcal/mol errors are acceptable. The LDF domain based scheme performs slightly better as it can drop around 10% more functions with similar accuracy. The NAF approach retains the least number of fitting functions with below 0.1 kcal/mol error. In conclusion, around 70–90% of the fitting functions can be dropped depending on the chosen algorithm with sub-0.1 kcal/mol errors in the reaction energies.

Importantly, these conclusions hold independently of the reaction type in these 4 test cases, including single, double and aromatic bond breaking. However, these reactions involve quasi-one-dimensional molecules in order to study embedding methods with limited system size. For this reason, it is important to assess the performance of our algorithms on real-life, three-dimensional target applications with larger active subsystem and realistic amount of bonds on the border of the active and environment regions, which is presented in the next sections.

### 4.2 Catalysis in Zeolite Cavity

The barrier height of the zeolite catalyzed reaction is assessed in Figure 4. In addition to the effect of the auxiliary basis reduction, the performance of the AO reduction with respect to the complete, reduction-free embedding calculation is also presented.

**Figure 4:**
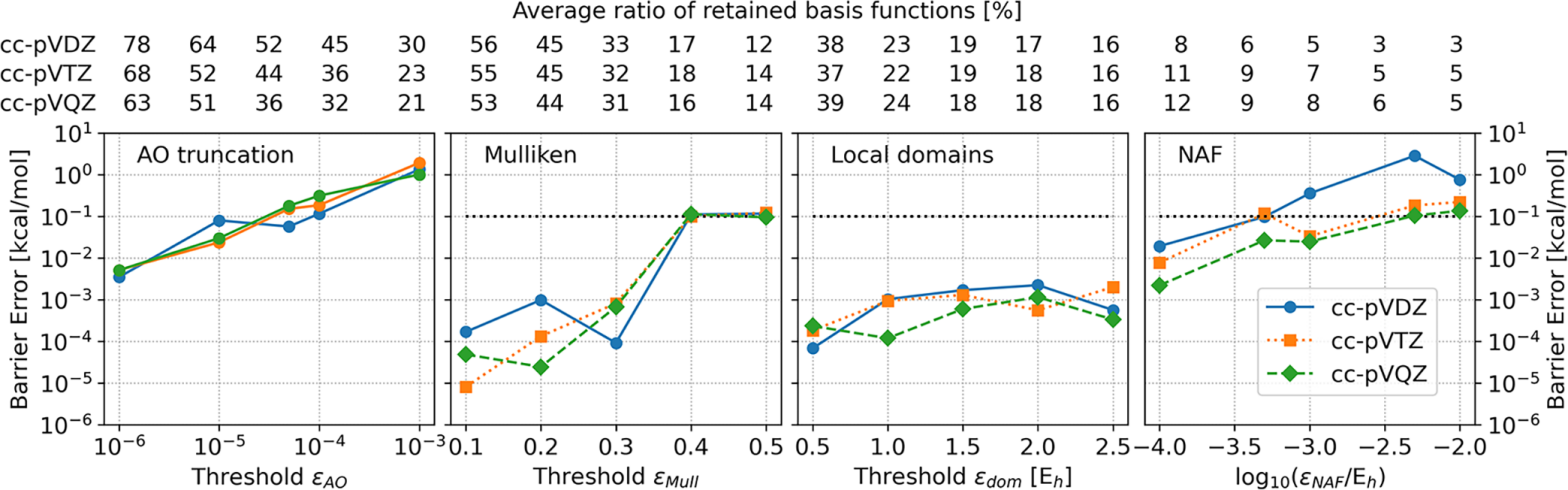
Barrier height errors for the zeolite system. The first panel shows the error of the AO reduction compared to approximation free embedding, while the other three panels show the error of the fitting basis reduction.

It is apparent from the results obtained with the cc-pV*X*Z (X = D, T, Q) basis sets that the errors of either the AO or fitting reduction schemes do not depend significantly on the basis set, as also observed for the smaller test molecules. The error of the AO reduction is around 0.1 kcal/mol for most threshold values. To get below 0.1 kcal/mol for all basis sets, one can opt for ε_AO_ < 10^–5^, retaining around 60% of the AOs.

Regarding the fitting basis reduction schemes, the generalized Mulliken charges approach gives somewhat higher, 0.1 kcal/mol error for looser (0.4–0.5) thresholds and a significantly smaller, 10^–4^–10^–3^ kcal/mol error for the tighter ones, while keeping around 30–40% of the auxiliary functions. The LDF domains based method performs more consistently. The errors are negligible, as they stay below 0.01 kcal/mol for all tested parameters and it only requires 15–20% of the original fitting functions. The NAF approach provides similar errors (around 0.01–0.1 kcal/mol) with a more compressed auxiliary space (below 10% of the original dimension) than with the other two algorithms.

### 4.3 Enzyme Catalysis

We show the effect of both the AO and auxiliary basis reduction on the intermediate, TS and product states in Figure 5. The results do not differ significantly, thus the basis reduction schemes can be safely applied for both barriers, intermediates and reaction energies. The results are similar to that obtained for the zeolite reaction, although the errors are slightly higher for the generalized Mulliken charge and LDF domain based methods, but still under 0.1 kcal/mol. On the other hand, the NAF-based approach becomes more accurate and the errors stay below 0.1 kcal/mol even with the loosest thresholds retaining 11–17% of the dimension of the original space.

**Figure 5:**
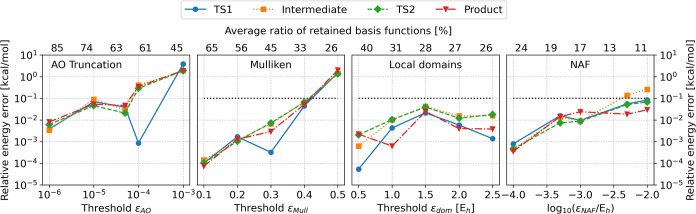
Relative energy errors for the enzyme reaction. For further details see the description of Figure 4.

The energy differences between the two barrier heights and between the intermediate and product sates are also accurate (see Figure 6). For the AO basis reduction, the errors are typically below or around 0.1 kcal/mol except for very loose thresholds. The fitting basis reduction schemes give errors below or around 0.01 kcal/mol in general. The LDF domain based approach performs the best, as the errors stay close to 0.01 kcal/mol for all tested thresholds. Thus, differences of energy differences often required for chemical conclusions are also highly reliable.

**Figure 6:**
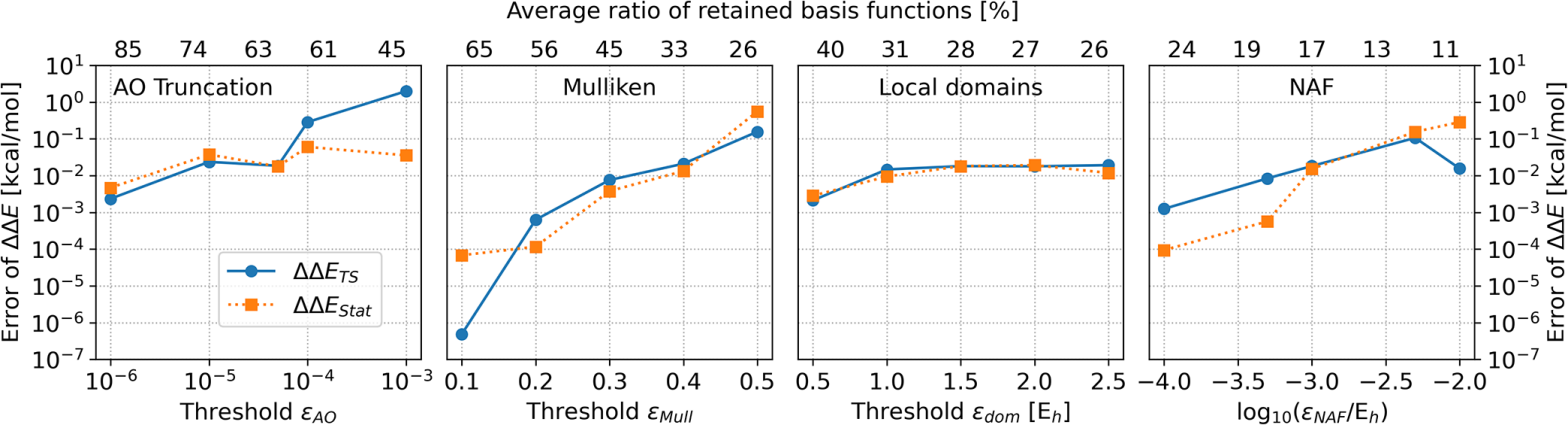
Error in the difference of the TS1 and TS2 transition states energies (ΔΔ*E*_*TS*_) and the intermediate and product stationary states (ΔΔ*E*_stat_). For reference, the energy differences calculated with basis reduction free embedding are 5.05 and 4.22 kcal/mol for ΔΔ*E*_*TS*_ and ΔΔ*E*_*stat*_, respectively. For further details see the description of Figure 4.

The number of retained fitting basis functions in the enzyme reaction is around 4–10% larger than for the zeolite with the same thresholds. It is the result of the relatively larger number of active atoms (87 vs 61). The NAF approach is still the most effective requiring about half as many fitting functions as the other two. Just as for the zeolite, the local domains based algorithm is more accurate than the generalized Mulliken charge based scheme if similarly sized fitting spaces are compared.

### 4.4 Organocatalysis

For the halocyclization, larger part of the substrates and the catalyst are required to be active (163 active MOs). The results are similar as for the zeolite and enzyme reactions (see Figure S3 of the SI). In brief, the AO basis reduction gives around 0.1 kcal/mol error at the 10^–4^ threshold value, where it drops approximately 33% of the AO functions. The generalized Mulliken charge based algorithm gives satisfactory accuracy of 0.1 kcal/mol below the threshold value of 0.3 and it discards 40–65% of the auxiliary functions. The local domain based algorithm is more accurate as the errors stay below 10^–3^ kcal/mol, while it keeps similar number of functions as the generalized Mulliken charge based method.

## 5 Timing and Primary Memory Requirements

To assess the performance of the AO and auxiliary basis reduction, runtime measurements were carried out on the three larger test systems with both integral-direct and the new in-core algorithm, using 16 cores (and 16 threads, with hyperthreading turned off) of an Intel Xeon Gold 6448H CPU. To provide multiple perspectives, the total speedup, the speedup in the high-level SCF and the ratio of high-level SCF and total runtime are plotted. The reference method is always the complete basis, reduction-free embedding calculation using integral-direct DF-SCF algorithm with the same basis set. That is, all reference hybrid and GGA computations are accelerated via DF and none of the timings reported in the paper were obtained via four-center algorithms. Timing measurements were performed in a production environment where competing computational load was present and not controlled.

Starting with the high-level SCF for the zeolite system, the AO reduction is around 2.6-times faster than the reference DF hybrid DFT (see Figure 7a). Using the in-core algorithm, the speedup can be increased to 12. Fitting basis reduction outperforms this with a speedup of around 20, even with the integral-direct generalized Mulliken charge or LDF domain based methods. The NAF and in-core LDF domain based schemes can reach speedups around 40–50 or even higher.

**Figure 7:**
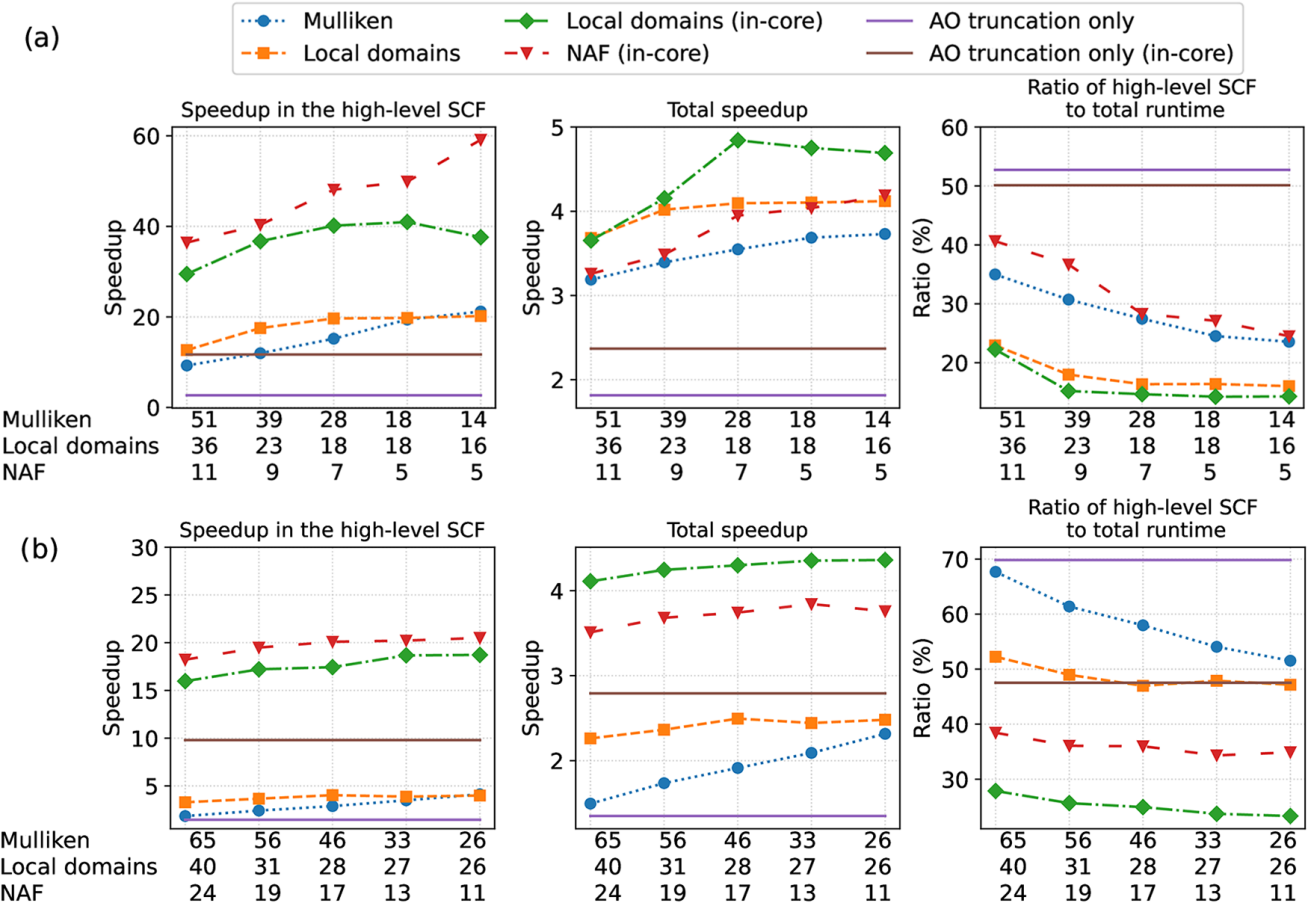
(a) Runtime data for the zeolite system using the cc-pVTZ basis set and (b) the enzyme reaction using the def2-TZVP-in-def2-SVP basis set. The *x* axis shows the ratio of the retained basis functions in %.

The speedups in the total embedding calculations (middle panels) are a bit smaller than 2 via the AO basis reduction alone, which can be improved to around 2.5 by the in-core algorithm. Turning on the fitting basis reduction schemes leads to better performance of 3–4 times speedups in general. Moreover, an about 5-fold acceleration in the total runtime is measured for the LDF domain and in-core algorithm. It is worth noting that the NAF approach is slower than the local domains based scheme, despite its higher level of compression. The reason is the relatively large overhead of the NAF (and thus also of the generalized Mulliken charge based) approach.

Finally, considering the ratio of the high-level SCF to the total runtime, it is 16–20% for the in-core LDF domain and NAF approaches and 25–40% for the integral-direct algorithms. Thus, we achieved that the most time-consuming step is now the low-level DF-based GGA calculation for the entire molecule, and further improvement in the high-level method will not result in significant additional acceleration.

In the high-level SCF calculation for the enzyme reaction (Figure 7b, left), the AO basis reduction achieves a speedup of 1.5, while the LDF domains and generalized Mulliken charge based methods show 4-fold acceleration. The in-core algorithms are significantly faster: for AO basis reduction a 10-fold speedup, and for fitting basis reduction around 15–20-fold speedups were measured. The smaller values compared to the zeolite system can be explained by the larger active MO list and thus the higher proportion of the kept basis functions.

The total speedups are similarly satisfactory as for the zeolite system. It is below 3 for the AO reduction only and integral-direct algorithms, while it is around 4 for the in-core fitting basis reduction methods. The high-level method takes around 50–70% of the total runtime for the integral-direct case, while the low-level DF-based GGA part dominates with 60–80% for the in-core algorithm.

For the halocyclization reaction, we get similar results as for the zeolite system (Figure S4 of the SI). With AO basis reduction alone, smaller than 2-fold speedups are measured. Using the auxiliary basis reduction too, around 10–12-fold speedup can be achieved for the high-level calculation and 6 times speedup in the total runtime. Here, the runtime of the high- and low-level methods are comparable. In the case of AO basis reduction only, almost 90% of the total runtime is spent in the high-level method, while with fitting basis set reduction, this is reduced to around 60%. The somewhat higher proportion of the high-level runtime is caused by the larger number of active MOs and the range-separated functional, for which two exchange matrices are built.

Based on the benchmark results above, we recommend threshold values of 5 × 10^–4^ E_*h*_ for the NAF, 2.0 E_*h*_ for the LDF domain and 0.3 for the generalized Mulliken charge based algorithms. These values provide both high performance gains and accuracy. The runtimes with these threshold are presented in Table 1. On 16 cores, starting from 527–1327 min without approximations, the new LESS approach achieves 10–32 min wall time. This is equivalent to around 2–8 CPU core hour for a single energy evaluation depending on the modeled system and DFT functional. The 10–32 min runtimes are comparable to the DF-accelerated GGA computations with full triple-ζ basis (12–23 min) and thus reaches the speed required for large scale sampling and (with forces under development) dynamics simulations in the future.

**Table 1:** Timing Data in Minutes for the Three Large Test Systems^a^

number of	zeolite	enzyme	halocyclization
atoms	171	238	179
active atoms	22	28	42
occupied MOs	646	466	352
active MOs	61	87	103

Wall Times in Minutes			
hybrid/TZ	1327	527	862
hybrid/TZ-in-DZ		139	273
GGA/TZ	12	15	23
GGA/TZ-in-DZ		6	19
hybrid-in-GGA	68	45	204
AO reduction	30	34	122
local domains	17	19	32
generalized Mulliken	19	24	40
local domains (in-core)	14	10	
NAF (in-core)	19	12	

a TZ labels full triple-ζ (cc-pVTZ is used for all zeolite computations), TZ-in-DZ means mixed def2-TZVP-in-def2-SVP basis set (used for all embedded enzyme and halocyclization reactions, including those where not labeled explicitly). All computations, including the non-embedded reference hybrid and GGA measurements, consistently employ DF. Thresholds: ε_AO_ = 10^–4^, ε_dom_ = 2.0 E_*h*_, ε_NAF_ = 5 × 10^–4^ E_*h*_.

The above timings clearly show that storing integrals in primary memory has an advantage over integral-direct calculations. However, it is not feasible to store in primary memory the complete set of three-center integrals even in sparse format for large basis sets and/or molecules. For typical active region sizes, the AO reduction can roughly halve the number of integrals, while the fitting basis reduction omits around 80% of the auxiliary functions. As a result, approximately 10% of the integrals is kept, which can very often be stored in the primary memory or at least on disk.

To show the viability of this idea, we find in Table 2 significant, 9–70 times primary memory savings for the zeolite and enzyme systems. Using the proposed approximations, the memory of a single workstation (i.e., here 20–40 GiB) is enough for our new in-core algorithm.

**Table 2:** Total Primary Memory Requirement of the In-Core Algorithm for the Zeolite and Enzyme Systems in GiB^a^

memory requirement [GiB]			
	zeolite		enzyme
	cc-pVDZ	cc-pVTZ	TZ-in-DZ
hybrid-in-GGA	192.8	1400^b^	181.8
AO reduction	66.2	220.0	105.1
LDF domains	11.4	40.5	28.2
NAF	4.1	19.4	19.9

a Thresholds: ε_AO_ = 10^–4^, ε_dom_ = 2.0 E_*h*_, ε_NAF_ = 5 × 10^–4^ E_*h*_.

b Estimated value.

## 6 Discussion and Conclusions

To summarize, we introduced and assessed local approximations to embedded subsystems, i.e., the LESS framework in combination with the Huzinaga embedding and DF algorithms. We implemented AO basis reduction and three new algorithms to select the important fitting basis functions in a Huzinaga or projection-based embedding calculation. The LDF domains based algorithm is accurate in all cases (below 0.1 kcal/mol error in both reaction energies and barrier heights), while it drops around 70–80% of the fitting function depending on the system. The NAF approach is also very accurate and can discard even more functions, approximately 80–90%. The LDF domains based scheme is usually the fastest of the three in total runtime as it drops a large portion of the fitting functions, while it has a negligible computational overhead. Moreover its generalization to, e.g., derivative properties is also relatively straightforward and it can be used together with integral-direct or in-core algorithms.

If one aims at an error on the order of 0.1 kcal/mol in relative energies, such as reaction energies and barriers (i.e., not effecting the overall accuracy of hybrid DFT), we recommend the thresholds of 5 × 10^–4^ E_*h*_ for the NAF, 2.0 E_*h*_ for the LDF domain and 0.3 for the generalized Mulliken charge based algorithms. The errors with these settings for the three larger demonstrative reactions are displayed in Table 3, as well as in Figure 8 for the barriers and in Figure S6 for the reaction energies. In all of these, comparisons are made to the complete DF-based hybrid DFT calculations (without embedding and basis reduction) using a triple-ζ quality basis set. The LESS approximated reaction energies, especially for the zeolite and halocyclization reactions, are even more accurate than the barriers. The errors in the absolute energies are also highly satisfactory, 10^–5^% and 10^–6^–10^–7^% for the AO and auxiliary basis reduction, respectively, and cancel excellently in energy differences (Table S1). The corresponding absolute and relative runtimes are collected in Tables 1 and 3, respectively. For example, comparing the first and last row of Table 3, we find that the DF-accelerated GGA and high-level hybrid DFT computations with the LESS approach are 46–111- and 27–93-times faster than the approximation free, DF-based hybrid DFT computation, respectively. Compared to their similar speed, the GGA and hybrid-DFT-in-GGA errors are 4.2–10.6 and 0.2–0.3 kcal/mol, respectively. Thus, we can conclude that practically hybrid DFT accuracy can be achieved (including all the embedding, mixed AO basis, AO and fitting basis reduction approximations), while the overhead of the hybrid DFT part is similar or less than that of a DF-accelerated GGA calculation for the full system (cf. Table 1). In other words, the total LESS hybrid DFT-in-GGA computation time is comparable to that of about two DF-GGA runs. The errors are mostly caused by the embedding and AO basis reduction, the fitting basis reduction introduces errors in the order of 0.01 kcal/mol. If motivated by tighter accuracy requirements in certain applications, naturally, all approximations can be systematically improved even further.

**Table 3:** Summary of Speedups and Errors [kcal/mol] with the Recommended Thresholds^a^

	zeolite		enzyme		halocyclization	
	speedup	error	speedup	error	speedup	error
**GGA/­TZ(-in-DZ)**	**111**	**4.90**	**86**	**4.23**	**46**	**10.63**
hybrid/­TZ-in-DZ			4	0.89	3	–0.32
hybrid-in-GGA	20	–0.14	12	0.35	4	0.36
AO reduction	45	–0.32	16	0.35	7	0.22
**LESS (LDF domains)** ^b^	**93**	**–0.32**	**51**	**0.34**	**27**	**0.22**

a For the zeolite, the cc-pVTZ basis set was used on all atoms in all cases. For the other two molecules triple-*ζ* quality basis set was used on the active and double-ζ on the environment atoms. The reference is the hybrid DFT calculation with triple-*ζ* basis set for both errors and speedups.

b The speedup values correspond to the in-core algorithm for the zeolite and enzyme systems, and to the integral-direct for the halocyclization.

**Figure 8:**
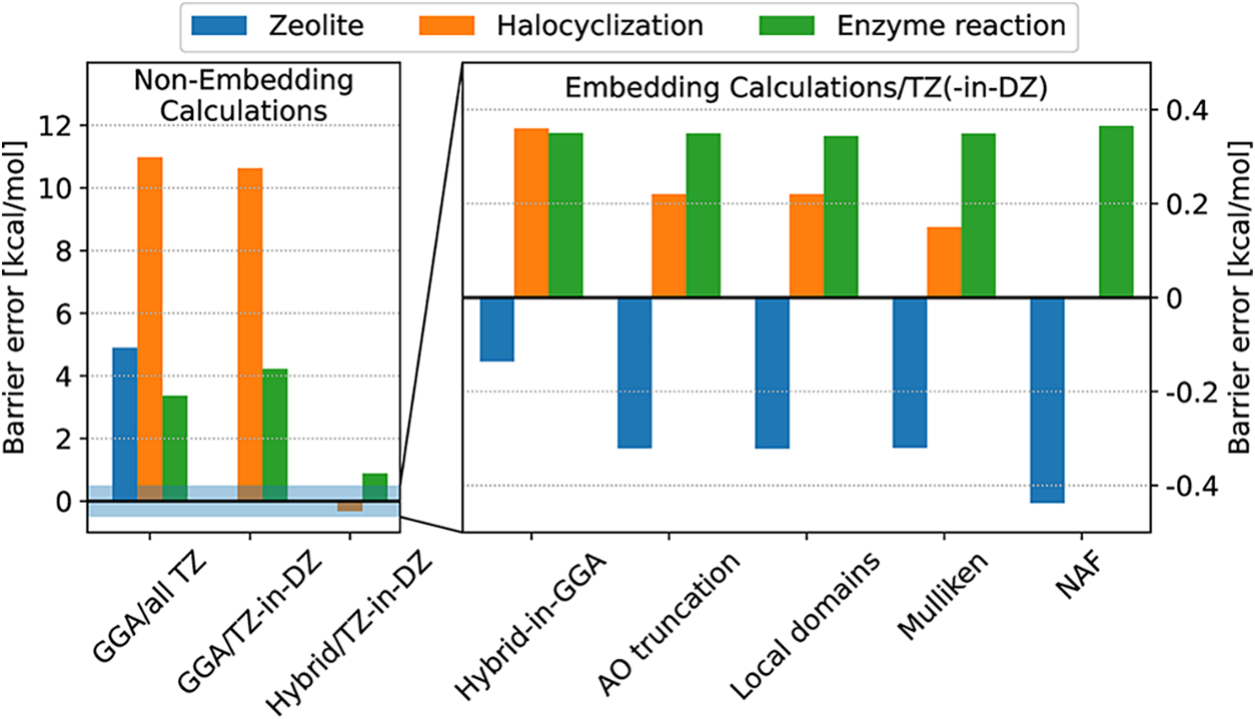
Summary of the accuracy of the different levels of theory for the three larger test systems with the recommended thresholds with respect to the hybrid DFT/triple-ζ results on the complete system. Barrier heights are shown here, while the corresponding reaction energy errors are given in Figure S6 of the SI. For the enzyme reaction, the barrier height of the first TS was used here.

The runtime acceleration is the combined effect of several techniques. On their own, each of the applied approximations (mixed basis, embedding, AO and fitting basis reduction) yield 2–4-fold speedups in the high-level computation. If used together, the effect of the approximations amplify each other and ca. 30–90-fold speedups are achieved. This performance is close to that of ONIOM (if one uses an active region that is tiny compared to the usual practice in ONIOM calculations), while the errors are decreased for these reactions by a factor of 10–50 compared to that of GGA calculations.

Due to this performance, we find the here introduced LESS framework highly promising for DFT-in-DFT embedding. We recommend as default the use of the LDF domain based algorithm, due to the advantageous properties, such as the high compression rate, the computational affordability and its compatibility with both integral-direct and in-core (or disk-based) algorithms. For these reasons, this LDF domain based algorithm is also the prime candidate for accelerating the analytic derivatives for embedding methods. The extension of the local embedded subsystem (LESS) framework is underway in our laboratory to not just DFT-in-DFT but also wave function-in-DFT type schemes including both single point energies and analytic gradients. Such developments should enable large scale structure optimization or dynamics applications including both proper environment effects and accurate treatment of reactivity.

## Supplementary Material







## Notes

Accuracy of the standard test systems; accuracy and timing data the halocyclization reaction; accuracy of total energies; OMP parallelization; accuracy of reaction energies; and example input (PDF)
The results obtained for the larger test systems (zeolite, halocyclization, enzyme reaction) are tabulated (XLS)
Cartesian coordinates and the applied active subspaces of the benchmarked molecules (XYZ)
