# Supplementary material for: Hybrid DFT Quality Thermochemistry and Environment Effects at GGA Cost via Local Quantum Embedding
Source: J Chem Theory Comput. 2025 Sep 29;21(19):9573–86. doi: 10.1021/acs.jctc.5c01121 (PMC12529908; doi:10.1021/acs.jctc.5c01121)
Supplement: Supplementary file 1 [file ct5c01121_si_001.pdf]

S1 Accuracy of the Standard Test Systems

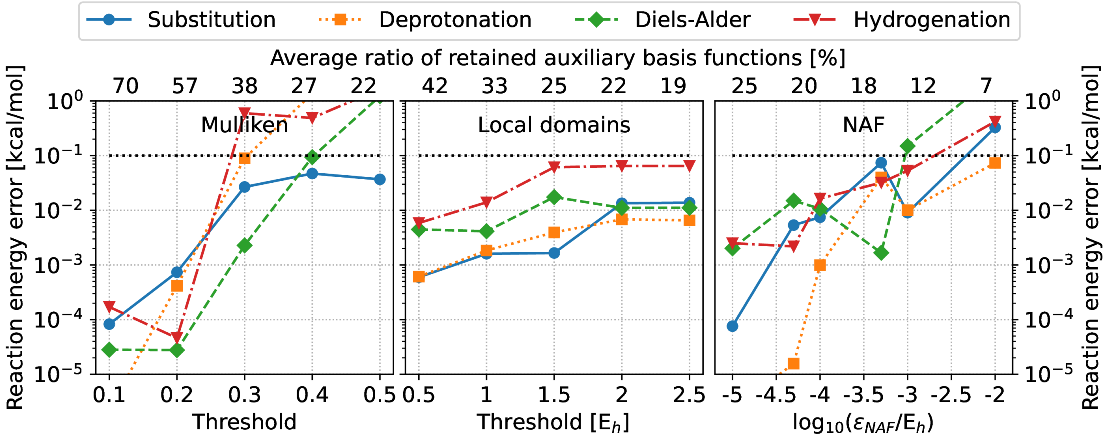

Figure S1: Reaction energy error of standard benchmark reactions (see Fig. 1 of the main text) using the cc-pVDZ basis set. The reference calculations use AO truncation only. The bottom x axis shows the applied thresholds, while the top x axis display the average ratio of kept fitting basis functions compared to the size of the complete auxiliary basis in %.

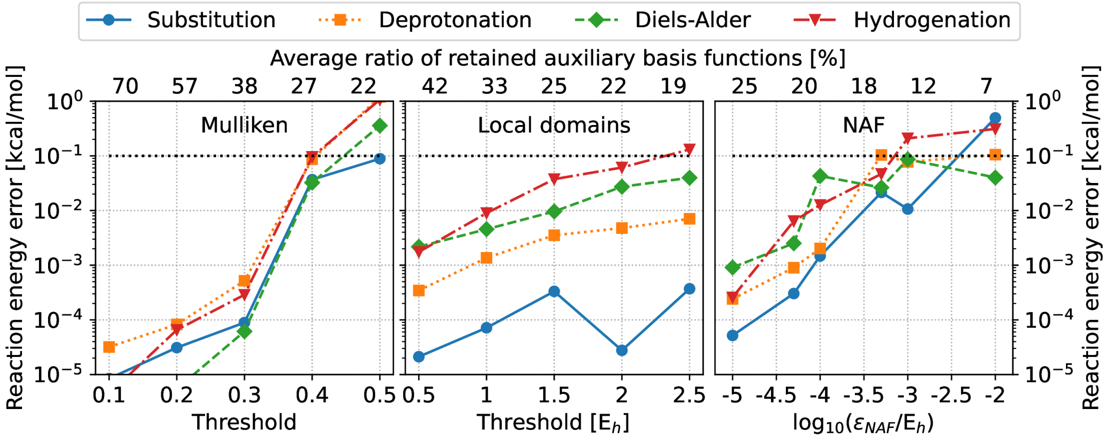

Figure S2: Reaction energy error of standard benchmark reactions (see Fig. 1 of the main text) using the cc-pVQZ basis set. The reference calculations use AO truncation only. The bottom x axis shows the applied thresholds, while the top x axis display the average ratio of kept fitting basis functions compared to the size of the complete auxiliary basis in %.

S2 Accuracy and Timing Data the Halocyclization Reaction

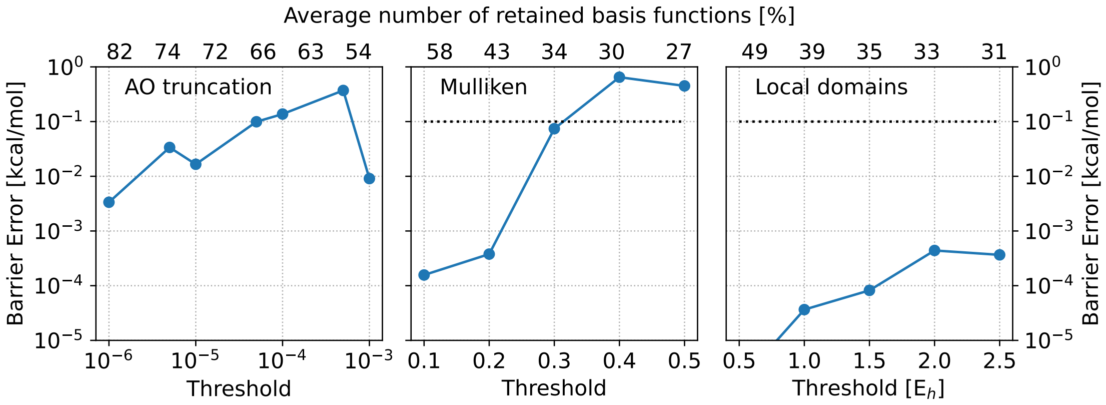

Figure S3: Barrier height error of the halocyclization reaction using CAM-B3LYP-in-M06-L embedding. The first panel shows the error of the AO truncation compared to approximation free embedding, while the rest three shows the error of the fitting basis truncation. The bottom x axis shows the applied thresholds, while the top x axis display the average number of kept fitting basis functions compared to the size of the complete auxiliary basis in %.

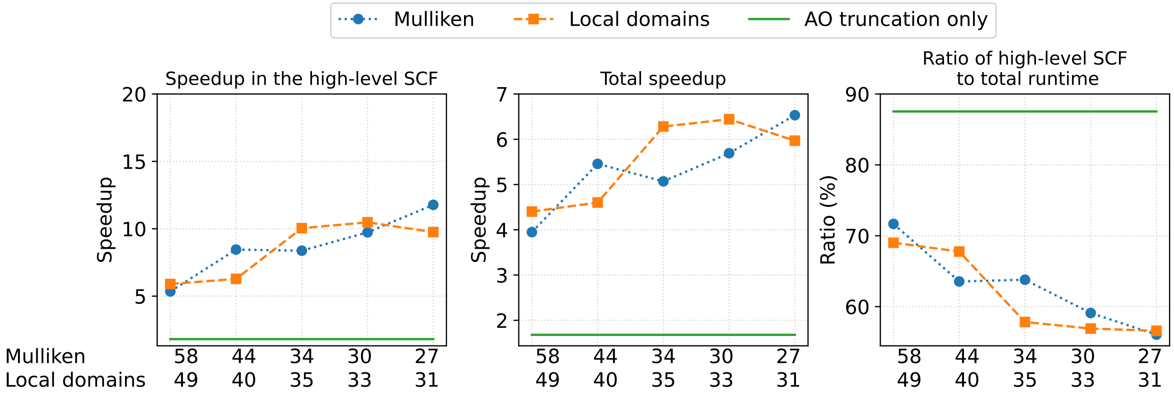

Figure S4: Runtime data for the halocyclization reaction with def2-SVP/def2-TZVP mixed basis set. The x axis shows the number of retained basis functions in %.

S3 Accuracy of total energies

The change in total energies (Table S1) caused by the AO basis set truncation is at the scale of a couple of kcal/mol in absolute terms, while the new auxiliary basis truncation approach is at least 1–2 orders of magnitude more accurate. The relative errors are in the 10^{-5}% and 10^{-6}%–10^{-7}% scale for the AO and auxiliary basis truncation, respectively. Most importantly, the active occupied orbital selection remains consistent along reaction coordinates, ensuring that most of the introduced basis set truncation error is canceled in energy differences.

Table S1: Total energies (in atomic units) and errors introduced by AO or auxiliary basis set truncation.

	Energy [E_h]	approximation error [E_h]	
structure	embedding PBE0-in-PBE	AO truncation ($\varepsilon_{\mathrm{AO}} = 10^{-4}$)	LDF ($\varepsilon_{\mathrm{dom}} = 2.0$)
zeolite RS	-18865.345379	0.004191	-0.000014
zeolite TS	-18865.306566	0.004485	-0.000013
zeolite PS	-18865.369623	0.004158	-0.000012
organocatalyst RS	-5016.629018	0.002118	-0.000005
organocatalyst TS	-5016.586536	0.002337	-0.000005
organocatalyst PS	-5016.670355	0.001862	-0.000005
enzyme RS	-7660.974594	0.004160	-0.000258
enzyme TS1	-7660.945937	0.004162	-0.000249
enzyme PS	-7660.965734	0.004714	-0.000251

S4 OMP parallelization

We analyzed the scaling of the overall LESS calculations for the reactant state in the zeolite cavity. The total runtime is 20.1 minutes, the program spends 5% of it in non-parallel modules, while the longer SCF cycles scale well, at least up to 8 threads. Better scaling can be expected for larger system size.

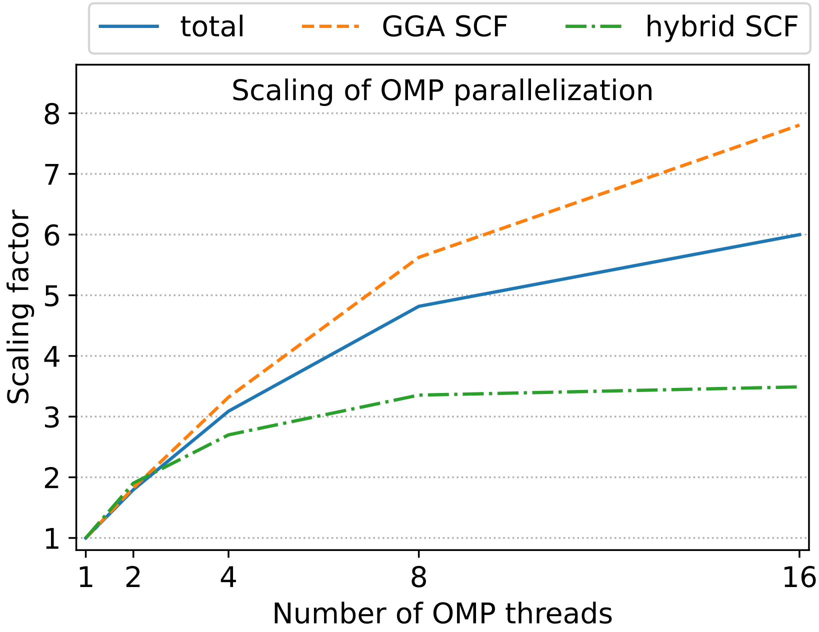

Figure S5: Speedup from OMP parallelization in the zeolite reactant calculation and its two main SCF iteration. Settings: PBE0-in-PBE/cc-pVTZ with $\varepsilon_{\mathrm{AO}} = 1 \times 10^{-5}$, $\varepsilon_{\mathrm{dom}} = 2.0$.

S5 Accuracy of reaction energies

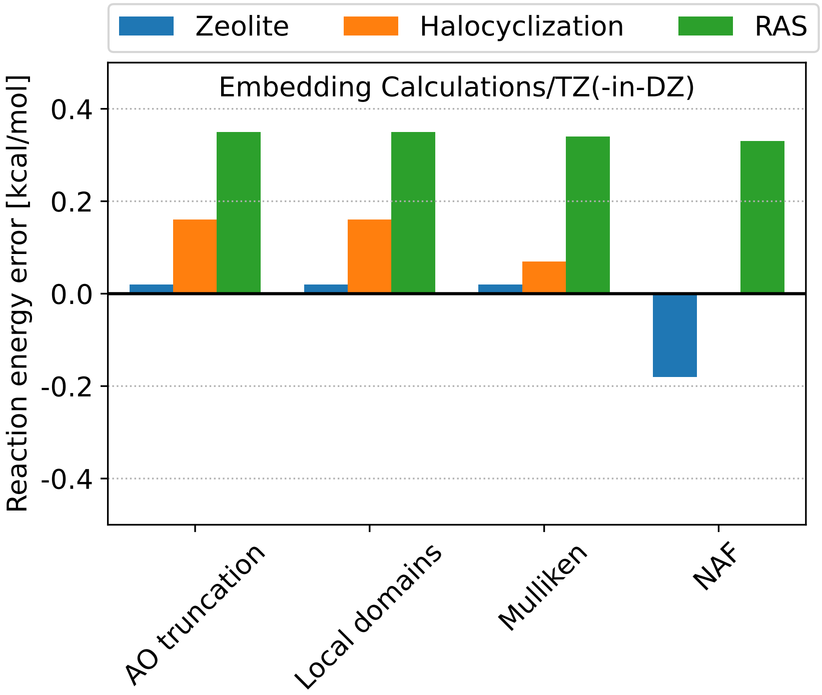

Figure S6: Summary of the accuracy of the different levels of theory for the three larger test systems with the recommended thresholds with respect to the hybrid DFT/triple-ζ results on the complete system.

S6 Example input

The following example demonstrates how the AO and fitting basis truncation methods can be used in the MRCC program package. The in-core algorithm can be turned on by the *incore* keyword. Additionally, the *scfalg* and *hl_scfalg* keywords have to be specified as well which control the low- and the high-level SCF algorithms, respectively. The in-core algorithm works only with disk-based SCF algorithms. Therefore, the *scfalg=disk* and/or *hl_scfalg=disk* options have to be specified as well, if in-core calculation is required. To run integral-direct SCF, *scfalg=direct* and/or *hl_scfalg=direct* have to be used.

The below example presents the input for a PBE0-in-PBE calculation for two water molecules, where one molecule is in the active space and the other constitutes the environment. The low-level PBE calculation uses integral-direct algorithm, while the high-level calculation is performed with the in-core algorithm. Both AO and fitting truncations are used. The fitting truncation uses the local domain based algorithm.

```
calc=PBE0              # Specifies the high-level calculation
basis=cc-pVDZ          # Specifies the basis set
ao_trunc=on            # Turns on the AO truncation
ao_trunc_tol=5e-5 # Specifies the AO truncation threshold
```

```
df_trunc=ldf        # Turns on fitting basis truncation using local domains
df_trunc_tol=2.0  # Specifies the fitting basis truncation threshold
scfalg=direct       # Integral-direct low-level calculation
dfbasis_scf=cc-pVDZ-RI-JK  # Specifies the fitting basis set

mem=500MB          # Specifies the memory MRCC can use
hl_scfalg=disk   # Disk-based high-level calculation
# Turning on in-core algorithm.
# It effects only the high-level calculation as
# the low-level SCF algorithm is integral-direct.
incore=on

orbloce=pm          # Pipek--Mezey localization for orbital selection
embed=huzinaga   # Huzinaga-equation-based embedding algorithm
1-3                 # Active atom list
pbe                 # Low-level method
0                   # Orbital selection (automatic, Mulliken charges)

geom=xyz            # XYZ geometry (in Angstrom)
6

H      -0.758      0.000      0.533
O       0.000      0.000     -0.057
H       0.758      0.000      0.533
H      -0.758     10.000      0.533
O       0.000     10.000     -0.057
H       0.758     10.000      0.533
```
